# “Getting on the same page” enhancing team performance with shared mental models—case studies of evidence informed practice in elite sport

**DOI:** 10.3389/fspor.2023.1057143

**Published:** 2023-05-30

**Authors:** Michael Ashford, Jamie Taylor, Jared Payne, Dom Waldouck, Dave Collins

**Affiliations:** ^1^ Grey Matters Performance Ltd, Stratford Upon Avon, United Kingdom; ^2^ Moray House of Education, The University of Edinburgh, Edinburgh, United Kingdom; ^3^ Faculty of Science and Health, School of Health and Human Performance, Dublin City University, Dublin, Ireland; ^4^ Insight SFI Centre for Data Analytics, Dublin City University, Dublin, Ireland; ^5^ Independent Researcher, Belfast, United Kingdom; ^6^ Gloucester Rugby Football Union Club, Gloucester, United Kingdom

**Keywords:** shared representations, team cohesion, collective decision making, coaching methods, rugby union

## Abstract

Within high performing, team invasion sports, collective decision making and coordination between teammates are essential characteristics. There is a wealth of evidence supportive of shared mental models as being an important construct to underpin team coordination. Yet, to this point, there is limited research considering the coaches' voices in the application of shared mental models in high performance sport, nor the challenges coaches face throughout the process. Given these limitations, we provide two case studies of evidence informed practice which privilege the voice of coaches who work in elite rugby union. In doing so, we aim to offer a deeper insight regarding the development, implementation, and continued use of shared mental models to enhance performance. Through these first-person case studies, we present the development of two shared mental models and the processes taken, challenges faced, and coaching methods used to underpin them. The case studies are then discussed with implications for coaches' practice supporting the development of their players' collective decision making.

## Introduction

Collective coordination and cohesion have been widely acknowledged as key characteristics of successful teams within elite sport (1, 2). Evidence derived from this field of research has led to practical recommendations which have suggested that team coordination and cohesion is best developed through the deliberate planning and strategizing of players collective behaviour to outwit and defeat opponents, largely supported by a deeper knowledge-of the game (3, 4). Furthermore, these approaches have led to a broad variety of applied implications. For instance, match strategy (5), Shared Mental Model's [SMM (6, 7)], Team Mental Models [TMM's (8)], and tactical frameworks (9). Importantly, all encourage the weighting and execution of specific roles and responsibilities along with exposure to conditions which guide players to behave in particular ways. Importantly, these plans may be centrally orchestrated or evolved through structured social interactions with, through and by the players (6, 10).

Perhaps the most prominent example of such processes within this field is the development and implementation of SMMs. SMMs have been defined as: “overlapping mental representations of knowledge by members of a team” that support team effectiveness (11). This suggests that, if team members have a shared and coherent understanding of the task that is to be performed and of the teamwork required to succeed, performance is likely to improve (12). The SMM construct has received wide support in group/team execution settings. A substantial volume of empirical data from multiple domains [e.g., (13–15)], points to its advantages. For example, in the context of sport, Richards et al. (6, 16) investigated the impact of SMMs on critical performance variables in hockey and netball respectively. Filho and colleagues have suggested that the development of SMMs—improve collective decision making in volleyball teams (17), shared regulation of behaviour in motor skill tasks (18), and serve to build team cohesion, collective efficacy, belief and buy-in to tactical approaches in football (19). Gershgoren et al. (20–22) tracked SMM development over the duration of a season in football, finding improved cohesion and creativity in collective decision making throughout competitive situations. Additionally, Giske et al. (23) explored SMMs within an ice hockey and handball team and suggested that they were an essential prerequisite to facilitate collective performance.

In extension of this work, Filho and Tenenbaum (8) suggested that SMMs feed into TMMs which both contribute to effective team coordination over time. The notion of TMMs implies that mirrored knowledge structures which overlap completely, are dysfunctional as they do not allow a weighting of individual or unit-based knowledge structures to overlay one another. Instead, Filho and Tenenbaum (8) suggest that, for a team of individuals to know what, why, when, where, how and utilise this knowledge to execute collectively, their mental models and labours should be distributed by reference to their roles and responsibilities, via units, leaders or key individuals. Furthermore, at the heart of this evidence base, Lines et al. (24), have recently published a systematic review and meta-analysis exploring the enhancement of SMMs and TMMs within sport and performance, made up of 6,209 participants and 1,912 teams, advocating for their evidence base and continued use. Consequently, at least from an empirical perspective, a weight of evidence suggests that the development, implementation, and continued use of SMMs and TMMs are useful components of optimal team coordination.

In addition to this breadth of evidence, some lay views have regarded SMM as being overly prescriptive or fixed (25). In turn, this may have reduced the potential the construct has to offer to coaches (26). Therefore, towards the aim of offering evidence informed examples of how SMM's may be employed by coaches, this paper presents first person case studies of applied practice and further recommendations for the enhancement of collective decision making in elite team sport. Accordingly, the later sections of this paper present how the idea was introduced and discussed with coaches, exemplifying the literature, ideas and approaches they interacted with. Subsequently, we present first-person case studies from two professional rugby union coaches working at the elite level of the sport, who have been supported to develop, implement, and use SMMs over multiple seasons.

### Developing a shared mental model

The authors of this paper make up the dynamic of the coach developer and coach alliance, ongoing since January 2021. The first, second and final authors make up a triad of coach developers who work with the third and fourth authors, both of whom are defence coaches in the English Premiership and previously the United Rugby Championship and French Top 14, the elite professional leagues of rugby union. It is important to note that both coaches, like many of their peers, spent significant time and effort aiming to generate shared understanding with their teams, without a good declarative understanding of the SMM construct. Thus, initially, the coaches demonstrated levels of tacit, and procedural knowledge, associated with many years of trial and error aiming to develop team coordination (27, 28).

In the early stages of the alliance Richards and colleague's empirical work exploring the development and use of SMMs within international level hockey, and netball were introduced to the coaches (7, 29). Through workshops, interactions with peers and one to one sessions with coach developers, coaches were given opportunities to challenge, understand, and conceptualise how SMMs would fit into their planning and practice over time. Central to these interactions were two key holistic models to develop a SMM to result in better collective performance (7). The first model offers a layered approach where the cognitions, situational factors, and performance setting, are proactively considered to develop the SMM. This is a five-phase non-linear cyclic approach with feedback and feedforward mechanisms for shared learning and development of team constructs: (1) the development of a performance vision (2) the sport specific skillset required to achieve this vision (3) tactical development (4) strategic development and (5) execution. Most prominently, it identifies how slow off-line thinking built over the macro, meso, and micro timescales, can be transitioned into execution of coordinated action under high pressure. Macro-associated timeframes refer to forecasting over a prolonged period (e.g., a four year cycle or season), meso-associated timeframes refer to medium term blocks where particular themes can be targeted (e.g., a 6–8 week block), whilst micro-associated timeframes refer to a particular moment in time [e.g., a week of training or session (27)]. The second model considers the employment of coaching methods which may be used during on-field and off-field training and competition environments (7). The central aspect of both models is the progression from a single coach's performance vision to an alpha version, which is a clear and coherent version of the performance variables in the coaches' own mind, to a beta version, which is a clear, coherent, but also an updated version of the original vision shared and agreed with the wider coaching and playing group (6, 7, 29).

Importantly, much of the commentary throughout this paper alongside the case studies build upon on the original works of Richards and colleagues (6, 7, 29). However, there isn't the space to get involved in a detailed overview of these concepts and language used, outside of the respective case studies [*but it is useful to be familiar with this work so please read* e.g., (6, 7, 29)]. Therefore, building on this work, the purpose of the paper is best captured through three distinct aims: (1) to privilege the voice of the coach to explore how ideas regarding SMM development derived from research have been adopted and applied in practice; (2) to acknowledge the day to day challenges coaches face in adopting and using SMMs (3) to make sense of the key ideas running through each case study to offer evidence informed recommendations to coaches who seek to improve their teams coordination and collective decision making. Additionally, and for complete clarity, this is not an attempt to offer a “best practice” approach to enhancing team decision making. Rather, these case studies share first person accounts of research informed practice (30) in the messy reality of high-performance coaching.

## Case studies

*Dom Waldouck—meso level SMM—defensive approach*

At the conclusion of the previous season, it was clear that our coaching and playing group had a fractured, rather than clear understanding of what we were trying to achieve when defending, driven by a combination of misguided belief and blissful ignorance. Honest and critical reflection unearthed a clear need to develop appropriate and genuine levels of understanding within the coaching and playing groups regarding what we were trying to achieve in defence. In essence, I had a clear understanding of the approach, but only some players had the clarity of understanding that I felt we needed to progress towards the upper part of the league table. Whilst I had a tacit understanding of the concept, once the structured approach to SMMs was introduced [cf. (7)], I identified four clear aims and objectives to achieve:

(1) To clarify a clear way of playing which represented the identity of the club with key language underpinning it.
(2) To share with, generate feedback from and create shared responsibility within the leadership group.
(3) To develop the whole squad's understanding
(4) To consolidate and deepen understanding for performance.

### Clarifying a clear way of playing

During the off season it was essential to weigh up where the playing group's defensive strengths and weaknesses lay, both individually and collectively. Furthermore, the “fractured understanding” which characterised our defence the season before was largely driven by a lack of buy-in from the wider playing group. The development of the new *performance vision* had to consider the player's characteristics biopsychosocially and generate complete support from the full playing group. An initial starting point was to bring our defensive way of playing closer to the desired social identity of the club, as a proudly working person's club where the fans value effort, commitment, and “tough” rugby. We felt this would suit our playing group and be appreciated by the fan base. We began by using the club badge and image of a *lion* to generate shared language for the initial performance vision.

This vision resulted in a discussion regarding an analogy of the lion's bite and behaviour preceding it, which included: (1) *position the block*—be in the best collective position (2) *jaws*—dictate where attacking players go and trap them; and finally, (3) *bite—*with maximum impact given the game situation. I also knew that this needed to be underpinned by a change to our collective mentality. Therefore, we needed to adjust the shared understanding of our mental approach and developed an alpha version of “a *fighter's mindset*” where players repeatedly get back in the game after making tackles until the ball is won back. In addition to these overarching principles, I identified the key technical and tactical requirements within defensive moments of the game, inclusive of movement behaviour, individual tackle technique and individual options available to win the ball back at the breakdown. This aimed to address one of our key weaknesses in the previous season, conceding unnecessary penalties as players lacked a deeper understanding of how their individual skillset fitted into the bigger defensive picture. Furthermore, we analysed players strengths and weaknesses defensively on an individual basis, which supported the alignment of the collective SMM and how it suited their unique skillset.

### Creating shared responsibility socially with and through a leadership group

At this point the vision remained an alpha version, conceived by me, but not yet shared, understood, or elaborated by the playing group. When I shared it with the rest of the coaching group, I wanted critique, comment, and debate. As a result of this, it was tweaked accordingly through mutual discussion. The changes were addressed in two ways, firstly rugby union is a strategically and tactically complex game. It requires the dynamic and successful knitting together of areas of performance to complement one another. For example, the game frequently transitions between attack and defence, but this is often made more complex as most teams will use specialist coaches for different areas. As a defence coach, I need to align with the lineout, breakdown, kicking, and attack coaches. Without a shared, coherent, and complementary understanding of what we are trying to do, there is a real possibility of the team causing problems for each other. For example, if we spend a lot of energy in attack in our own half, this will significantly compromise our energy and ability to defend for longer durations if the ball is turned over. Therefore, I needed to ensure that the new defensive approach aligned with other aspects of the team style.

The second purpose was to share ideas with the coaching group and to have these ideas challenged for the purpose of co-construction. This process extended beyond an opportunity to offer critique of the alpha version, but importantly, built a deep understanding regarding how we intended to defend mutually. The other coaches were then able to reinforce defensive ideas and more easily develop understanding of transition moments between the different areas of the game.

Following this, I and the other coaches selected a leadership group made up of defensive players who we felt would offer a good blend of different roles in the defensive SMM, and who had already demonstrated the desired technical, tactical, physical, psychological, and social features of the performance vision. This began as a core group of six players, spread across positions to facilitate knowledge transfer in the wider playing group, the positional units, and sub-units. This was later supplemented by a variety of other players who entered and exited the group throughout the season to deepen their understanding, without adding unnecessarily high cognitive load throughout the training week due to other commitments.

The performance vision was offered to the playing group to clarify the tactical components and the common language used to support player understanding. Like the coaching group, the players offered critical thoughts on the vision, and through listening, constructive conflict (at times) and extended discussion we came to a clear agreement of what we wanted to build across the playing group to be successful. During this time, there was a reciprocal knowledge exchange between the coach(es) and defensive group, where increased responsibility was expected of players. Following this, the defensive group undertook weekly refinement exploring a different approach in pre-season and in-season. In pre-season we followed the same process, beginning the training week by reviewing our progress in relation to our overall performance vision. In-season, we used game day minus five (GD − 5) to debrief the previous week, review adjustments, what we had learned, and to identify intentions for the coming week. For the most part, this was all derived from our performance vision. On GD − 4, the defensive group would collectively plan the week and session content. This primed players to drive themes and messages to the wider squad. Socially, this increased the likelihood of messages being received by the playing group, if they were receiving them from leaders within the playing group as well as their coaches. Thus, through this social diffusion, players became aware of the purpose of on and off-field learning activities, which provided increased opportunities to use knowledge on the pitch by reflecting in action, engaging in self-regulated learning, and hopefully, metacognition. Finally, on GD − 2 the defensive group, would work with me to develop the game preview meeting which aimed to highlight the key elements of our approach to beating our opponent (see Table 1). Often, I would use an individual player to lead this meeting, further promoting player engagement and responsibility in the SMM and the social dynamics behind its delivery and success.

**Table 1:** An example breakdown of the slow off-field content broken down throughout a week.

Game day-6	Game day-5	Game day-4	Game day-3	Game day-2	Game day-1	Game day
*Coach planning*	*Learning day:* Debrief of previous week with defensive leadership group. Adjust key parts of defensive SMM. Shape clear intentions for the week	*Collective planning day:* Plan weeks content & session content in relation to intentions. This is done with and through the leadership group.	*Coach planning*	*Develop preview of opponents:* leadership group lead on final preview meeting of opponents. Identify key elements of SMM to outwit opponent defensively.	*Final preparation*	*Performance*

### Developing the playing group's understanding

As the performance vision was shared between coaches and the leadership group, it supported further knowledge exchange to the wider playing group. It also seemed to increase shared responsibility across the defensive player group. Initially, we employed slow off-field and deliberate pedagogic approaches [cf. model 2—(7)], often beginning with slow walk and talk sessions to clarify player understanding. Other slow off-field and deliberate methods included team meetings, weekly reviews/previews of training, looking at game footage of other teams, or previous footage of our own defensive performances. This footage was combined with a range of coaching approaches, including open discussions, and direct instruction and debate, to increase the awareness and understanding of the players as they were asked to solve problems in small groups using classroom settings. Initially, players' problem solving was heavily scaffolded where I framed a particular problem, introducing where they should be looking, what they should be looking for, and why. With shared understanding increasing over time, scaffolds were gradually removed leaving players to identify, acknowledge, and solve the problems presented. For instance, in the later stages, I used a clip from the previous season where an opposition attack broke us down after several phases, then used a variety of divergent questions to ask how we could have solved the problem and why. Only then did I use our performance vision (and the layers of detail underpinning) to check for coherence with the SMM to see what areas of shared understanding needed refining with new solutions. In addition, when new concepts were introduced to the playing group, we also used a range of bridging methods designed to transfer thinking from slow off-field to fast on-pitch. These drill practices were often low in physical fidelity, especially given the high physical demands of our sport, but we always aimed for conceptual fidelity. We were using slow thinking, followed by low level drills to embed the understanding of the performance vision during more game like practice or games.

### Consolidate and deepen understanding

To further reflect, consolidate and deepen understanding around our SMM, we used nested planning (27) over the duration of the season. We were consistently engaging in critical reflection to monitor, test and tweak our way of defending contingent on our overall intentions and performance against specific opponents. As such, although the core principles of the performance vision remained the same, we would adjust through the season to test new ideas or with specific opponents in mind. As a result, I often planned blocks of games in advance, looking at how many weeks ahead where new knowledge would need to be first introduced, then weighing up the extent to which it would be appropriate for our next opponents.

As players' understanding of the defensive performance vision improved, their individual mental models became distributed to specific roles and responsibilities. Based on feedback, it seemed that players increasingly understood why classroom and on-field activities were important. Additionally, the coaching and leadership group shared, corrected, reinforced, and offered feedback on the relationship between the vision, the situation, action, and terminology used by players. Mondays were often labelled a *“learning day”*, which went beyond walking through new plays and priming the week ahead. We also spent a lot of time extensively debriefing the previous game, not just treating it as something to get done in a few video clips. The intention for the week was established between the coaching group, discussed with the defensive playing group, and introduced through a slow off-field discussion and meeting. Then we typically provided a low physical demand, isolated practice, that would specifically target that aspect of performance. As the week progressed faster on-field and representative activities would be put on for the players to recognise key cues, actions, and responses to typical and atypical game situations. These fast and often more physically demanding sessions stress tested the performance vision, to assess whether individual mental models, roles and responsibilities, knowledge structures, and technical/tactical actions were shared amongst the whole playing group.

### Challenges and methods to overcome them

There were several challenges that were faced throughout the process of developing an effective SMM. First, was the initial process of going from *my* alpha version to *our* shared beta version of performance that was agreed by all. There were areas of the original defensive approach that players believed weren't feasible. However, because I encouraged the coaching group and playing group to challenge the original vision, points of agreement and disagreement were discussed, debated, and resolved. Second, to ensure understanding of the playing group, all coaches needed to hold a shared and coherent understanding of our defensive SMM. Therefore, my challenge was to develop the coaches' understanding as much as possible to make sure we were disciplined and coherent with our language and messaging when communicating with players. By developing this shared understanding through debate, the increase in touch points and voices promoting key messages seemed to be a significant factor in supporting the development of understanding across the group. Third, by selecting the small defensive player group, that left 40 players who were excluded from key defensive conversations. More players in the group would have diffused levels of engagement and responsibility, hindering the quality of conversations.

Fourth, the off-field, classroom-based preview/review sessions evolved over time. Initially they were driven by me, standing at the front, and giving information to the players. This quickly shifted to a variety of pedagogic approaches, one being a ‘flipped classroom’, where footage was sent out before the session to prime the players, so discussion was immediate rather than watching lots of video clips in the meeting. Fifth and finally, an ongoing struggle for all rugby environments is the volume of information and consequent cognitive load experienced by players. At times, we became guilty of chasing too many ideas and concepts which watered down the impact of the week's learning opportunities. Critical reflection, and coach and player feedback highlighted that we made the mistake of getting too broad, offering too much volume and detail. These reflections resulted in a desire to have a clear intention for the week, which we hoped would improve role clarity, prime players learning experiences and support us to scaffold them through interventions where needed.

*Jared Payne—micro SMM—specific strategic problem*

It was during my employment at a previous club, where I first encountered the SMM concept. It was there that we already had a well-established performance vision/way of playing based on what we called our “Big Rocks” or technical and tactical principles, which had been developed over three years. The principles included: (1) *spacing* between defenders, (2) *scanning* of opponents, the threats they pose and the ball, (3) when to *defend the ball vs. when to defend the space*, (4) *load balance* which refers to the ability for us to change direction at speed and (5) *tackle entry* which includes the pace, direction, and height in which the tackle is executed. Although these ideas were already being used, deepening my declarative knowledge of the SMM construct has supported clarity, coherence, and collaboration within my practice. As a result, I am working through a balance of slow off field environments, with collaborative previews/reviews, defensive meetings, conversations, and video analysis (individually & collectively) between coaches and players. Alongside that, we use varied, fast on-field coaching methods to test for and prompt new learning opportunities. We have progressed to a deep understanding of the performance vision that is shared amongst the playing group with strong buy in.

One issue raised by our performance analysis department was that our collective decision making was considerably worse when things weren't going well for us in game. For instance, when we conceded a penalty, we tended to follow this up by conceding another. It was clear that specific match outcomes, both positive and negative, had an immediate adverse effect on player behaviours which could snowball. For example, in a game we had taken a 17-point lead in the first twenty minutes and were dominant, but we conceded a single penalty which seemed to spiral us into a 10-minute period where momentum was lost, culminating in the scores being level as we continued to concede penalties. As a group of coaches, we decided that we needed to add another layer to our SMM that enabled players to collectively respond adaptively to both positive and negative game moments. Thus, and once again building on the model from Richards et al. (7), led to the generation of four clear objectives to follow;

(1) *Increasing players' awareness of momentum*—support the playing group to recognise clear positive and negative changes in momentum during competitive performance.
(2) *Developing a shared and coherent intervention*—change the thoughts, feelings, and emotions of the playing group following these outcomes.
(3) *Employing on-field coaching methods*—change players actions and behaviour to keep positive momentum and break negative momentum.
(4) *Monitoring and measuring success*—performance analysis and off-field strategies to reflect on how momentum was managed throughout competitive performance.

### Increasing players' awareness of momentum

Initially, it was important to understand how different challenges were impacting our individual and collective decision making and what this meant to every player within the squad. To begin this process, we supported the players to recognise the challenges and the resulting behaviours in response to positive and negative match outcomes on both sides of the ball (attack and defence). Working with the performance analysts, we decided to create a momentum chart of efficiency that captured positive, negative, and neutral outcomes as they progressed throughout the duration of competitive games. From there, we presented this to the players to evidence the relationship between keeping momentum, breaking negative momentum, and match outcomes (e.g., scores for/against, line breaks for/against, turnovers for/against, penalties for/against). Periods of positive momentum were presented to the players visually as tall blocks of game moments which we termed *mountains,* which supported the opportunity to create an analogy with the players of taking the opponent to the top of the mountain. This was then extended beyond recognising a representation of defensive momentum alone. We asked the playing group to understand why, enabling role clarity and individual awareness of where improvements could be made. Here slow, deliberate off field methods were used in game reviews, where the momentum chart would be shown, and relevant footage identifying where it had been maintained or lost. We then used this as a stimulus for reflective discussions within game reviews, where clips were presented to the players for commentary and deeper discussions regarding collective courses of action were initiated. This created a useful tool to support player's recognition of possible challenges to momentum, albeit in an off-field setting.

### Developing a shared and coherent intervention

With an enhanced awareness of the problem amongst players and coaches, we then needed to build from off field understanding to shape on pitch solutions. I considered two coaching methods, firstly, staying within the bounds of our existing way of playing, to develop players' understanding of tactical solutions to best mitigate the strengths of opposition on a week-to-week basis. Second, recognising that at some point we would always come under pressure and concede points, and so, develop a tool for players to recognise how to mould their thoughts, feelings, and emotions positively to regain momentum. Conversely, to recognise when they had momentum, what created that momentum and how they could keep it.

By this point, our performance vision and way of playing had been well established over the previous three seasons. We had shared and coherent knowledge structures, with the tactical principles, common language, perceptual strategies, clarity on roles and responsibilities, needed in the game. Building on this, we deliberately put substantial levels of responsibility on the playing group to refine and adjust roles and responsibilities on a week-to-week basis. This meant that the defensive leadership group (*n* = 12–14 players) were heavily involved in the reviewing of previous games and previewing of opponents from week to week. On GD+1 our performance analyst shared the statistics and momentum chart from our previous game with the leadership group, whilst I gathered ideas and discussed their thoughts and how they *felt* on the pitch. Where appropriate, the players would identify video clips that captured these points for discussion. I would also send the leadership group an information pack of statistics and video clips which captured the technical and tactical strengths and weaknesses of our next opponent. Using this information, alongside the opportunity for debate between players and coaches, the leadership group would utilise key considerations from the review and preview to identify key technical/tactical solutions that would best mitigate our next opponents' strengths and expose their weaknesses. In turn, this would allow us to set the themes and agenda for the week leading into the game. The leadership group would then present this information to the wider playing group within a team meeting to frame the week's focus.

The second coaching method included a process of co-constructing a tool where players could intervene to break negative momentum and keep positive momentum within the game. An alpha version was developed which centred on three key considerations: (1) returning the playing group to an emotional baseline despite the previous events in the game, (2) hot debriefs which highlighted how the game was going and finally (3) a rapid summary of what needed to come next. In presenting this to the senior playing group, it was decided that team huddles would be the best place to employ this type of tool. These occur during penalties, tries scored or conceded, natural breaks in play such as substitutions and breaks in play where physios were on the field treating injuries. Debate and discussion led to the players wanting a procedure to follow to achieve the previous considerations, which added another layer to our SMM. First, partly driven by the wants of the players and to define a set point for players to listen to each other, coordinated deep breathing was employed before any player could speak. This breathing wasn't used for any physiological benefit, but instead as a prompt for reflection on the momentum of the game and metacognitive awareness. Following this, the players used their self-developed acronym SCOPE—where senior players from the leadership group would present key information including the score, game context, the opportunities available to them, key plays (tactics) that would expose their opponents' weaknesses and a summary of how to best execute this (see Table 2). Where appropriate, coaches and staff members who were on the pitch as water carriers would provide messages to the senior players before they began this process. With me being in the stand communicating key messages via the radio, this again reinforced the necessity for all coaches and staff to be on the same page, both with the process that was being used by the players, but also the SMM.

**Table 2:** SCOPE—developing a SMM for player led team huddle processes within competitive games to successfully navigate ebbs and flows in game momentum.

Score	What’s the score in the game and what does this mean for us?
Context in the game	How is the game going so far and how long is left on the clock?
Opportunity	Where are we strong vs. where are we weak? How do we ensure that we emphasise where we are strong in the way we’re playing?
Plays	What tactical solutions i.e., plays will allow us to emphasise our strengths? What do we need to avoid?
Execution	What do we need to do to execute this more effectively?

### Employing on-field coaching methods

Following the development of these coaching methods it was essential to relate these slow and deliberate concepts which existed hypothetically, to the fast reality of coaching practice. On GD − 5, following the player and coach led preview of our opponent, the defensive strategy and SCOPE SMM would be walked and talked through on the pitch, establishing individual roles and responsibilities, our opponents' key players, and informational cues that would inform our decision making. During these low intensity, low fidelity, training sessions, we mainly used a combination of instruction and guiding questions to check for the playing group's understanding of roles and responsibilities. As the week progressed, we used higher fidelity, more representative practices, where we designed sessions with the need to counter breaks in momentum, or to keep momentum that had already been built. Some examples of this included purposeful challenges, using game rules and practice conditions, to either reduce or increase the likelihood of success or instructing the attack to change their tactical approach. This provided the senior players with an opportunity to employ the SCOPE process in response.

Over the season, we fluctuated our priming of these on pitch activities, where sometimes the senior playing group were informed that these scenarios were likely to occur, giving them the opportunity to plan. At other times, we tested by just throwing it at them and creating surprising circumstances. Instead of closely monitoring what was said in the huddles, we decided as a coaching group and leadership playing group that SCOPE should occur in authentic game like situations without coach involvement. The rationale being the need for players to practice what we expected them to do in competitive games. Consequently, the players took responsibility for managing the SCOPE SMM and multiple opportunities were offered to engage them in critical reflection during and post session to explore opinions regarding how the huddles went, and how the management of momentum followed within the session. Formally, these reflective conversations would take between 5 and 10 min during the session, but informal conversations with players happened frequently throughout the week. Both formal and informal reflective conversations asked players to share their thoughts on the body language, modes of communication, mindset, and response to the huddles, and consider the feedforward of where they could be improved and why.

### Monitoring and measuring success

The continued use of the momentum chart in correlation with specific match outcomes allowed us to identify moments where momentum was kept for prolonged durations, when momentum was lost, but also when it was regained. Reflective conversations with senior players over WhatsApp, phone calls, or in person, also supported us to understand how the SCOPE SMM was perceived by the group and its effect within competitive games. However, these measures and conversations also gave the coaching group an opportunity to critically reflect on our game plan heading into the game and our wider SMM at the time.

### Challenges and methods to overcome them

The first challenge was multidisciplinary in nature as it was essential that the SCOPE process was embedded into sessions where stress testing of our SMM took place [cf. (9, 31)]. To overcome this challenge, it was necessary to engage all staff members (coaches, S & C coaches, heads of performance, medical team) in the careful planning of these instances so that any injury or fatigue factors could be mitigated. Similarly, priming the leadership group that SCOPE would be necessary within sessions the day before was also a key method, as it meant that player initiation of SCOPE became more time efficient within sessions.

Secondly, regular group reflection had already become central to the development of our defensive SMM over the previous three seasons. However, when you ask players to reflect in game, under competitive situations, this adds a level of difficulty with the necessity for a deeper level of reflection (i.e., metacognition). Richards et al. (29) identified the difficulties in asking senior athletes to verbalise their perceptions, thoughts and feelings and suggested that such processes should initially be scaffolded heavily to remove distracting influences. Indeed, the applicability of early development of metacognition has already been highlighted [e.g., (32)]. Therefore, it was essential that the playing group understood their role in the SCOPE process, and this meant only some players could speak to minimise extraneous cognitive load and finally, to ensure that the wider playing group took an action point to apply in performance.

### Sensemaking

Both case studies present real-life, first-person accounts of how elite professional rugby union coaches have successfully developed and implemented a SMM to improve team coordination, playing strategies, and tactics. Both coaches made use of the processes advocated by Richards et al. (7) and have offered insight into the steps, coaching methods, and challenges, which make up their case studies. We next offer sensemaking of these case studies by offering key applied implications for coaches that can be considered in a conditional fashion for their own context [cf. (28)]. Furthermore, it is important to restate that these are not case studies which offer “best practice” but rather, evidence-informed methods in the “swampy lowlands” of practice (33, 34).

## Coach driven performance vision

First, both case studies suggest that beginning with an ideal end in mind is a useful starting point in the development of an effective SMM. This must be a plausible performance vision which captures the true complexities of the current/or future game (6, 7). For instance, Lara-Bercial and Mallett (35) suggested that elite serial winning coaches have the ability to envision future changes to the nature of their sport and in support of other literature, were able to use this to develop novel, innovative, solutions (36). Both case studies present original performance visions that have been driven by their own context and playing group, but also using SMMs as a mechanism to embed these visions across the team that they coach. A coach's ability to anticipate future trends in the game are a critical feature of practice for both high performance and talent development coaches (7). As such, given the pace of change across team sports and the necessity for innovation in practice, SMMs are a useful and impactful solution for team sport coaches to navigate such challenges as these changes occur. Indeed, by developing shared constructs and critical thinking, such approaches can prime team members for future and essential reflection and adaptability (37). Additionally, however, and reflecting the work of Richards and colleagues (6), both case studies also describe the essential *psychosocial* aspects of SMM development, stressing the need to develop, ensure, and ongoingly promote, group buy in and, ideally, ongoing contribution.

## Integration of the SMM

In addition to the necessity of a coach leading the initial framing of a performance vision, the case studies suggest that co-construction alone is insufficient to support genuine shared knowledge. Although numerous sources have recommended that coaches should enable athletes to lead and take responsibility for their way of playing (38, 39), these case studies highlight both the depth of knowledge, creativity, and mental projection necessary to achieve this. Additionally, the case studies present the social complexities of attaining a coherent and successful SMM. There is a need for the alpha performance vision to be subjected to a process of co-construction and constructive conflict (11). This should see the alpha version exposed to critique from other coaches and the playing group (40, 41) and the beta version developed with *significant and demonstrable* input from those concerned.

Reflecting this group approach, methods should acknowledge the validity of multiple sources of knowledge across and between the staff and playing groups (42, 43), with diffused responsibility allowing for individual perspectives to be moved into collective focus (44). The necessity of constructive conflict means that knowledge is not only distributed, but truly shared (11, 45). Therefore, coaches should aim for honest group discussion and constructive conflict, generating a zone of uncomfortable debate [ZOUD (11)], regarding what is best for the collective (46). Understanding and meeting the players wants is area of difficulty for coaches managing the delicate balance of promoting autonomous decision making, but within the boundaries of appropriateness for a group of 40 or more players. Thus, whilst athlete driven practice may be desirable, the ability to envision and develop SMMs at this level of performance requires a significant level of prior expertise. Indeed, given that the buck stops (at least primarily) with management, this seems to be an invitation to potential disaster!

In both the cited cases, coaches aimed to navigate and orchestrate this complexity using carefully selected cultural architects [cf. (47)] who may otherwise have proven difficult in the implementation of the SMM had they not been engaged in a collective approach or been made responsible for supporting knowledge transfer in the wider playing group. Therefore, the social complexity of this process and the selection and agreement of who decides what, in this context, was performance focused. This integrated process will enable coaches to create and lead on tactical approaches to specific opponents or add layers to the teams collective SMM (6, 48). Varying degrees of supportive autonomy offered to a playing group is perhaps best captured by the number of players included in each coach's leadership group within their case studies (i.e., 5/6 vs. 12–14). The former employed a very small number of players, but also described the initial use of more directive coaching methods to implement a new way of defending, whilst the latter described adding additional detail to their SMM which had been established, grown, and adapted with a group of players over time (29).

In addition, at the HP level, coherence of a SMM is necessary across the coaching, playing, and performance, staffing group. In this sense, the development of SMMs are a truly interdisciplinary problem, requiring tight integration of input (49). In both these case studies, it is easy to see how the work necessary to generate a SMM in a single area could significantly inhibit progress in another. For example, if physical demands of the defensive SMM required something different to that of the attack.

The generation of the performance vision and management of the refining process also puts significant stress on the declarative understanding of the coach (50). For example, breathing in team huddles has become “fashionable” in elite rugby union, with players and coaches copying the practice of other teams. Notably, however, in each case study the coaches asked players to use coordinated breathing for a specific and deliberate purpose of refocussing the playing group (28, 36). In these instances, the coaches’ underpinning rationales and forms of conditional knowledge that underpinned their coaching methods are perhaps more important than the delivery of the method itself, put simply, the “wise know the whys” (51).

## Transfer of knowledge to performance

Both case studies suggest the advantages of shared understanding in enhancing team performance. We strongly suggest that coaches who support their co-coaches and playing group to build a deep game understanding will serve to better create shared perceptual strategies (3, 52), coordinate the use of a common language (7), develop coordinated responses to typical and atypical situations (53) and also collectively infer when tactical changes are required in game when a plan may not be working (53). Though not explicitly used as a theoretical basis for these case studies, these ideas seem to align with notions of predictive processing [cf. (54)] and active inference [cf. (55)], where players solve problems in an automatic, controlled, and meshed, fashion, driven by an internal predictive model (56). Therefore, effective SMM's are likely to support the development of players knowledge of and in the game (*i.e., what are we seeing/hearing/feeling? What does it mean? What comes next?*). In turn, the team will be better equipped to identify areas of strength and weakness in theirs and their opponents’ approaches; recognise trends in opponents or referee's decision making; support players to recognise, predict, infer, communicate, adapt, and execute, coordinated actions (41, 53). It is also important to note that this level of understanding is more multi-faceted than simple forms of shared knowledge between teammates, which have previously been discussed as a limitation of SMM development (57–59). Instead, strategic, metacognitive awareness and deeper declarative knowledge supports coordinated recognition of when adaption is required [cf. (60) and increases the likelihood for innovative and creative technical/tactical solutions within competitive performance (21).

## Enabling flexibility

We also hope that these case studies provide practical and conceptual clarity for operationalisation of SMMs. SMM's aren't fixed, or prescriptive top-down directives of behaviour. Instead, the introduction, development, and continued employment of SMMs captures a process which is flexible to the context, coaches, players, and temporal performance demands as evidenced through each case study (6). A SMM is not a static set point, instead it should be used to critique, and learn from, enabling reflection during and after performance. This fluidity was demonstrated in the conditional nature in which coaching methods were employed by both coaches to develop and implement their SMMs (29). The coaches both employed a balance and blend of slow, deliberate off-field and faster on-field learning activities (7) in their practice which were planned for in relation to the week's guiding intentions. Additionally, their coaching approach and learning activities ebbed and flowed between direct instruction, and knowledge transfer (61) to more orchestrated, game like activities which served to stress-test the SMM under representative conditions. The cases also serve to highlight opportunities for the role of the performance analyst's in going beyond analytics, moving into a more proactive positioning, and becoming embedded as part of a coaching group.

Finally, the case studies also highlight the utility of the SMM construct, building on previous applications in the literature which has focused on tactical and strategic elements of performance [e.g., (1, 7)]. Here, the construct has been used to generate both interaction based SMMs and relative understanding of the skills of different team members (62).

## Conclusion

In extending the work of Richards and colleagues (6, 7), the purpose of the paper was to privilege the voice of the coach, to explore how ideas regarding SMM development derived from research have been adopted and applied in practice. The case studies offer pragmatic, first-person accounts of the utility of the SMM construct in the high-performance team sport coaching environment and hopefully dispel misinterpretations of SMMs as being overly prescriptive. Further, acknowledging the day-to-day challenges coaches face in using SMMs; and to make sense of the respective case studies to offer evidence informed recommendations for team coordination and collective decision making. Accordingly, we would encourage readers to trial the ideas in their own practice, reflecting the advice of Schön that reflective practitioners be experimenters [cf. (63)].

Furthermore, we believe this further informs the body of evidence substantiating the development, implementation, and use of SMMs within teams (7, 53). Finally, by summarising the underpinning evidence, processes, coaching methods, challenges, to overcome them within the SMM process, we hope this paper acts as a resource which stimulates academic critique, discourse, and further developments within coaching practice.

## Data Availability

The original contributions presented in the study are included in the article, further inquiries can be directed to the corresponding author.
